# Dissecting the Mechanisms Underlying Substrate Recognition and Functional Regulation of O‑GlcNAc Cycling Enzymes

**DOI:** 10.1021/acschembio.5c00633

**Published:** 2025-10-15

**Authors:** Ziyong Z. Hong, Jacques Lowe, Jiaoyang Jiang

**Affiliations:** † School of Pharmacy, 5228 University of Wisconsin−Madison, Madison, Wisconsin 53705, United States

## Abstract

Protein O-linked β-*N*-acetylglucosamine (O-GlcNAc) modification, known as O-GlcNAcylation, is an essential post-translational modification (PTM) that plays critical roles in regulating various cellular processes, ranging from transcription and signal transduction to protein degradation. O-GlcNAcylation levels are dynamically regulated by a single pair of human enzymes: O-GlcNAc transferase (OGT) and O-GlcNAcase (OGA). Dysregulation of O-GlcNAcylation has been implicated in many diseases, including cancer, diabetes, neurodegeneration, and cardiovascular disorders. In the past decade, remarkable progress has been achieved regarding the structures of OGT and OGA proteins, as well as a series of innovative chemical and engineered tools that inhibit or induce the activities of these enzymes. While initial studies mainly focused on the catalytic domains of these enzymes, recent research has begun to uncover the structural and functional roles of non-catalytic regions. Notably, domains such as OGT’s tetratricopeptide repeat (TPR) and intervening domain (Int-D), as well as OGA’s stalk domain and pseudo histone acetyltransferase (pHAT) domain, have emerged as critical contributors to enzyme functions. This Account discusses recent progress in studying these essential enzymes, especially highlighting their unique structural features and intrinsic flexibility as potential mechanisms underlying their substrate recognition and functional regulation. New perspectives and research directions are also discussed. Such information is expected to facilitate the rational design of novel modulators of OGT and OGA to enable more specific functional control and potential treatment of disease.

## Graphical abstract

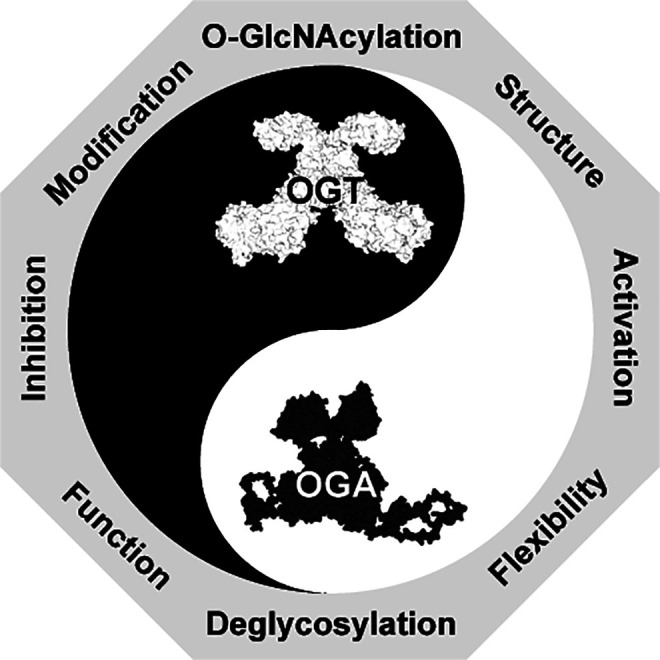

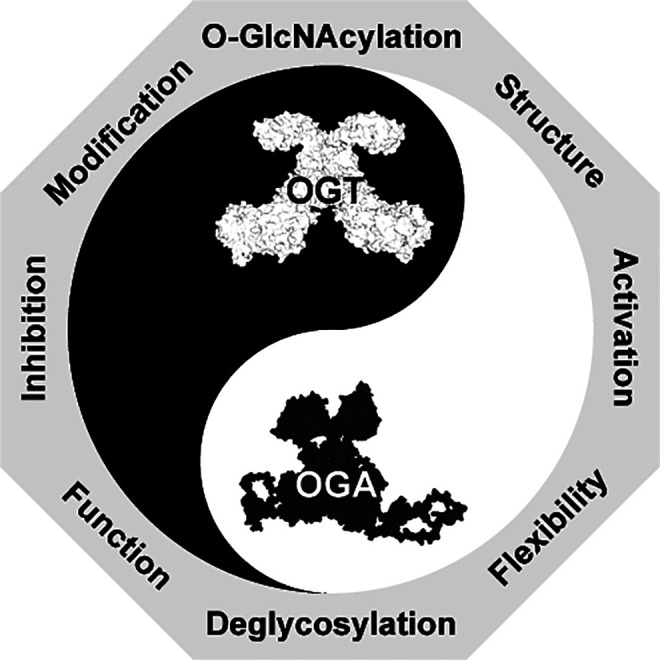

## 1 Introduction

Protein O-linked β-*N*-acetylglucosamine (O-GlcNAc) modification (O-GlcNAcylation) is a dynamic and essential post-translational modification (PTM) that regulates a broad range of cellular processes including gene expression, signal transduction, and cell cycle^1–4^ (Figure 1A). Interestingly, the regulation of O-GlcNAcylation is orchestrated by a single pair of human enzymes: O-GlcNAc transferase (OGT), which transfers a single sugar *N*-acetylglucosamine (GlcNAc) from the sugar donor, UDP-GlcNAc, to serine and threonine residues of protein substrates; and O-GlcNAcase (OGA), which removes the O-GlcNAc modification.^5–8^ Unlike classical glycosylation, which is primarily found on membrane/secreted proteins and involved in protein folding and trafficking, O-GlcNAcylation operates mostly within the intracellular environment (e.g., nucleus, cytoplasm, and mitochondria) and affects protein’s activity, stability, localization, and interaction networks.^9–12^ This single pair of O-GlcNAc cycling enzymes, OGT and OGA, modify thousands of protein substrates without a conserved sequence motif at the O-GlcNAc modification sites.^13^ The mechanisms of these enzymes in substrate recognition and functional regulation have been long-standing mysteries in the field and have become popular areas of investigation.^14–21^ Another unique feature of O-GlcNAcylation is its reversibility, allowing for fine-tuning cellular processes in response to external and internal stimuli, underscoring its critical role in maintaining cellular homeostasis.^1,22^ These O-GlcNAc events reflect a delicate balance between the enzymatic activities of OGT and OGA, impacting nearly every aspect of cellular biology and human health including immune response and organ functions. It is therefore not surprising that dysregulation of O-GlcNAc levels is correlated with the development of many diseases such as cancer, diabetes, neurodegeneration, and cardiovascular disorders.^23–28^ Hence, a deeper understanding of OGT and OGA, including their protein structures, substrate recognition, and functional regulation, is critical for dissecting disease mechanisms and developing novel modulators for potential therapeutic applications.

**Figure 1:**
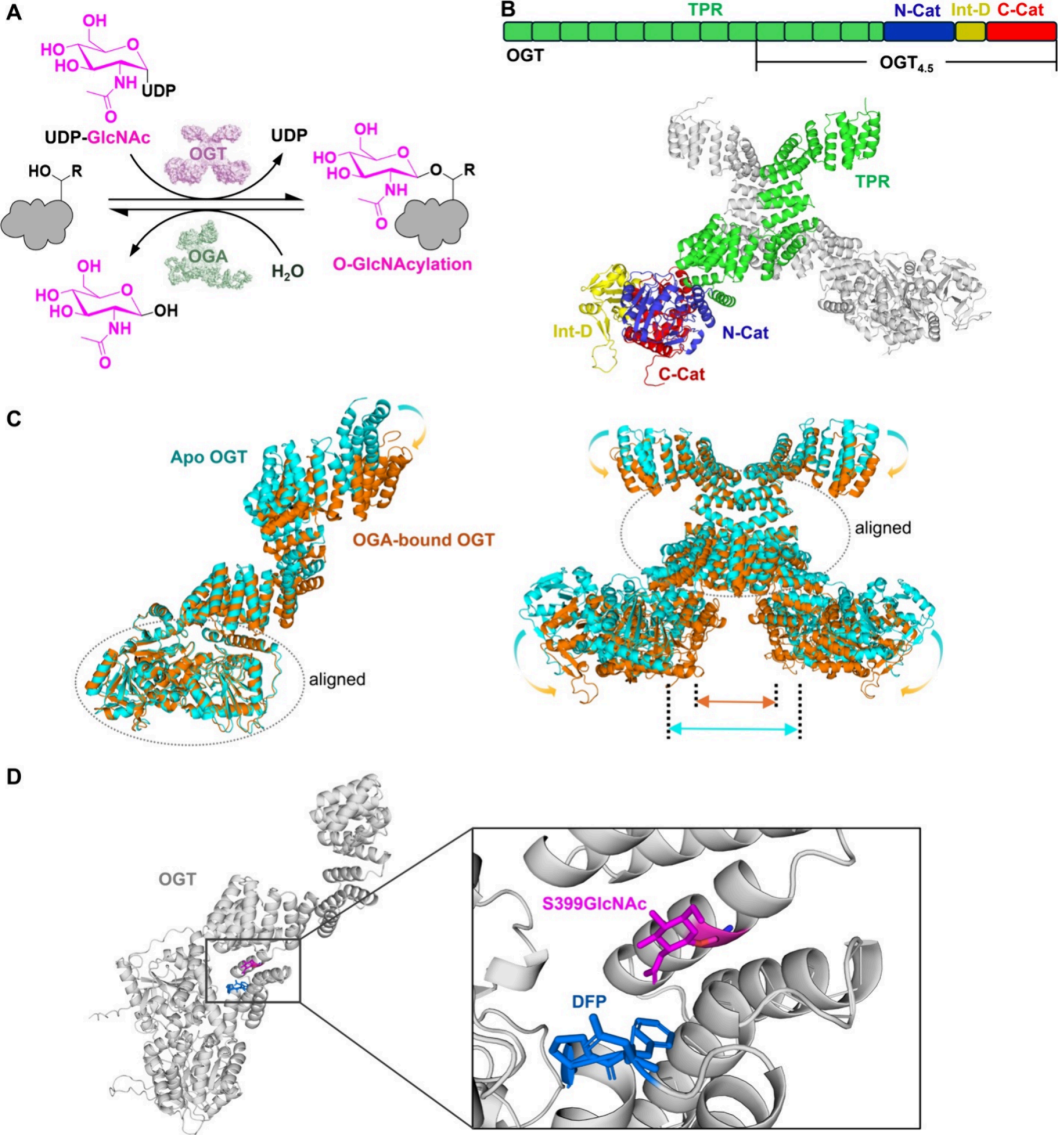
**A**. Reversible protein O-GlcNAcylation catalyzed by O-GlcNAc transferase (OGT) and O-GlcNAcase (OGA). **B**. (Top) Domain architecture of human OGT and the crystallization construct OGT_4.5_. (Bottom) OGT dimer modeled from cryo-EM structure (PDB 7YEA). **C**. Comparison of OGT conformations in apo form (cyan) and OGA-bound complex (orange). (Left) OGT monomer is shown. (Right) OGT dimer is shown. Alignment regions are highlighted by dashed circles. **D**. The relative positions of O-GlcNAcylation of residue S399 and the DFP motif on OGT as predicted by Alphafold3 (ref 29).

In the past decade, substantial advancement has been made in deciphering the structural and functional properties of OGT and OGA.^24,30–35^ However, obtaining a complete understanding remains challenging. OGT and OGA have evolved unique structural features that contribute to their substrate recognition and functional regulation. This allows these essential enzymes to bind a wide variety of substrate and nonsubstrate proteins, and carry out their multifaceted cellular roles with remarkable functional plasticity that is not yet completely understood. Given the broad range of substrates and functions of O-GlcNAc cycling enzymes, the principles governing their substrate recognition and functional regulation could be complex and go beyond their active sites.^13^ Based on the current knowledge of the structure–function relationship discovered from us and other groups, we propose a perspective that the catalytic domain of each OGT/OGA enzyme works in concert with structured non-catalytic domains and flexible regions within the enzyme to regulate ligand binding and functional plasticity. In this Account, we will discuss each OGT and OGA enzyme from three main aspects: briefly summarize the field’s understanding of their protein structures and functional regulation, highlight recently discovered novel allosteric sites and regulatory mechanisms arising from structural, biochemical, and post-translational modification (PTM) studies, and describe a few representative chemical biology tools for investigating their structures and functions. Lastly, we will discuss potential research directions to advance our understanding of the regulatory mechanisms of O-GlcNAc cycling enzymes and the implications of related knowledge in developing novel modulators and potential therapeutics.

## 2 O-GlcNAc Transferase (OGT)

### 2.1 A Brief Overview of O-GlcNAc Transferase (OGT)

OGT is a multidomain enzyme that catalyzes the O-GlcNAcylation on thousands of proteins.^5^ This enzyme is composed of an N-terminal tetratricopeptide repeat (TPR) domain and a C-terminal catalytic region, which is split into two catalytic lobes (N-Cat and C-Cat) by a unique intervening domain (Int-D)^15^ (Figure 1B). Three isoforms of OGT with varying numbers of TPRs have been identified: nucleocytoplasmic OGT (ncOGT) with 13.5 TPRs, mitochondrial OGT (mOGT) with 9 TPRs, and short OGT (sOGT) with 2.5 TPRs.^36^ Since ncOGT is the most abundant and best characterized isoform with demonstrated significance in a broad range of cellular processes, this Account will focus on the ncOGT isoform (termed “OGT” hereafter). Prior structural studies have shown that the TPR domain of OGT folds into a super helical structure and possesses a series of asparagine residues (termed “asparagine ladder”) aligning inside the TPR lumen.^37^ Mediated by the asparagine ladder, the TPR domain can accommodate substrates ranging from short peptides to flexible regions of large proteins.^38^ This ladder binds to substrates mainly through backbone interactions, acting as a general scaffold for OGT–protein interactions and substrate recognition.^38–41^ Following biochemical and mass spectrometry (MS) studies further discovered that a few residues in the TPR lumen display differential roles in binding to distinct protein substrates and may also contribute to OGT’s substrate specificity (see below).^39,40^ Intriguingly, the TPR domain is essential for OGT dimerization, which can enhance the enzymatic activity and substrate diversity. The initial view of the catalytic region of OGT was obtained from the crystal structures of truncated OGT (called OGT_4.5_ containing 4.5 TPRs, Figure 1B) in apo form and in complex with sugar donor (UDP-GlcNAc) and a peptide substrate.^15^ These structures revealed that UDP-GlcNAc binds specifically to a few residues in the OGT active site, while the peptide substrate lies on top of the sugar donor, making primary backbone interactions with OGT active site residues.^38^ This binding mode explains the absence of a consensus sequence motif at the O-GlcNAc modification sites. Working in concert with the TPR domain scaffold, OGT’s catalytic domain can modify serine and threonine residues across a wide array of substrate proteins and peptides. Surprisingly, a novel regulatory site has been recently discovered in the OGT’s intervening domain (Int-D).^42,43^ This cryptic domain separates OGT’s catalytic region into two lobes (N-Cat and C-Cat, Figure 1B) and is uniquely found in metazoan OGTs. While Int-D has been hypothesized to influence OGT’s cellular localization, substrate selection, and regulatory factor binding, direct experimental evidence has only recently emerged (see below). These exciting new discoveries show that OGT can recognize specific peptide motifs through the novel regulatory site in the Int-D, which plays critical roles in substrate/nonsubstrate protein binding, O-GlcNAc regulation, cell metabolism, and nutrient sensing functions of OGT, underscoring its potential as a functional hotspot for future chemical biology development.

### 2.2 The Structural Flexibility of OGT

While the structures of majority part of the TPR domain (containing 11.5 TPRs) and a truncated OGT (OGT_4.5_) were solved by X-ray crystallography over a decade ago,^15,37^ the structure of full-length OGT was only resolved recently thanks to the advancement of cryogenic electron microscopy (cryo-EM).^16^ This structure confirmed the dimerization of OGT mediated by the TPR domain. More recently, a cryo-EM structure of full-length OGT in complex with an OGA polypeptide shows that substrate-bound OGT adopts a different conformation from its apo form^31^ (Figure 1C), illustrating the structural flexibility of TPR. Another interesting observation from this complex structure is that the catalytic domains of dimeric OGT converge, while the N-terminal TPR repeats shift apart (Figure 1C). This motion indicates the elastic and dynamic nature of the TPR superhelix, even within OGT’s generally structured architecture. Moreover, the crystal structures of OGT_4.5_ in complex with different ligands revealed hinge-like movements between the TPR and catalytic region.^15^ This finding was also supported by molecular dynamics (MD) simulations.^15^ More recently, simulation studies further suggest that the TPR domain functions as an elastic nanospring, with single-point mutations capable of altering its global dynamics.^44^ Taken together, the flexibility of OGT may function as an additional layer of regulation for substrate recognition, and the ample conformational space enables accommodation for a broad range of substrates. In the future, it would be interesting to explore if the different OGT conformations are a consequence of ligand binding via conformational selection or an induced-fit mechanism, which underlies many biological processes. Integrating structural and biochemical data with molecular modeling is promising to derive structural and dynamic models of how substrates are recognized and processed by OGT. This will provide critical information to understand how OGT regulates its activity toward thousands of protein substrates to achieve selectivity.

### 2.3 Post-translational Modifications (PTMs) of OGT

Diverse post-translational modifications (PTMs) have been identified on OGT under various conditions and may regulate the cellular localization and functional activities of this essential enzyme.^45,46^ Nonetheless, the structural consequences of many PTMs on OGT and the exact mechanisms by which they affect function remain underexplored. Here, we will discuss the PTMs that have been studied in greater detail in this context. First, OGT can install the O-GlcNAc modification on its S399 residue, which has been shown to enhance the nuclear localization of OGT.^47^ Structural analysis shows that OGT S399 resides in a loop between two helices near the DFP region (residues 461–463) (Figure 1D). It is likely that the O-GlcNAcylation of S399 exposes the DFP region of OGT, promoting its interaction with importin α5 for nuclear transport. In addition to O-GlcNAcylation, OGT can be phosphorylated by a few different kinases. The phosphorylation of OGT T444 residue by AMP-activated protein kinase (AMPK) modulates OGT’s catalytic activity, subcellular distribution and substrate specificity.^48^ This phosphorylation affects O-GlcNAc dynamics and downstream signaling (e.g., mTORC1 activation and AMPK suppression), linking nutrient sensing to metabolic regulation. Under cold stress, the protein kinase PERK phosphorylates OGT, despite that the specific sites have not been identified.^49^ This PERK-phosphorylated OGT demonstrated enhanced activity to glycosylate TOM70 on its S94 residue. The O-GlcNAcylation of TOM70 directly enhances MIC19 protein import into the mitochondria and promotes cristae formation and respiration during stress adaptation. In another example, GSK3β phosphorylates OGT at serine residues S3 and S4, enhancing its enzymatic activity.^50^ Despite that the mechanism remains unclear, this finding was supported by mutational studies in which alanine substitutions at OGT S3 and S4 residues (S3A/S4A) prevented activity enhancement, while aspartate substitutions (S3D/S4D, mimicking phosphorylation) increased OGT activity. Although aspartate and glutamate mutations have been widely used as phospho-mimetics to investigate the roles of this modification, caution should be applied because in some cases these simple mutations cannot retain the same function as phosphorylation.^51–53^ To date, the crosstalk between O-GlcNAcylation and phosphorylation has been extensively studied for some proteins but not for OGT itself. This could be an interesting topic for future investigation. Besides O-GlcNAcylation and phosphorylation, other post-translational modifications have also been found on OGT, including acetylation and ubiquitination.^46^ Of particular interest, a recent study reported that FBXO31 induced ubiquitination and degradation of OGT protein, which can regulate O-GlcNAc homeostasis.^54^ Future studies are expected to reveal new insights into additional PTMs on OGT.

### 2.4 Chemical Biology Tools for OGT

Over the past few years, many chemical biology tools have been developed for OGT, which have been reviewed in excellent reports.^55–61^ Below, we will highlight a few representative developments that accelerate the investigation of OGT’s structure and function.

OGT plays crucial roles in human health and disease, but its substrate recognition remains poorly understood. To help address this challenge, our lab reported a series of conceptually novel chemical tools (called GlcNAc Electrophilic Probes or GEPs) that allow rapid mapping of key structural features of OGT for substrate recognition.^35,62^ Our rational GEP design was inspired by the observation of a unique cysteine residue (C917), in the OGT active site, in close proximity to the *N*-acetyl group of UDP-GlcNAc (PDB 4GZ5). Our GEPs contain a suitable electrophilic functionality (e.g., GEP1 in Figure 2A) that can react with the nucleophilic side chain of OGT C917, while retaining the ability to O-GlcNAcylate substrates similarly to the native sugar donor UDP-GlcNAc (PDB 5VIF). By introducing an azide handle to GEP1, the new probe (GEP1A) enables rapid detection of modified proteins (OGT and its substrate proteins) through a click chemistry-based fluorescence assay, allowing for a direct readout of altered substrate protein binding. Leveraging this unique assay, we performed a systematic analysis of the OGT TPR domain and discovered a number of important residues.^62^ Interestingly, distinct protein substrates (e.g., Nup62 and OGA) demonstrated differential preferences for TPR residues, suggesting specificity and plasticity of OGT’s substrate protein binding, which requires further investigation. More importantly, our GEPs help address the challenge of genuine OGT substrate identification for transient or weak interactors. Remarkably, we found that GEP1 (or GEP1A) can cross-link OGT in situ with a variety of peptide and protein substrates^35^ (e.g., PDB 5VIE), which are compatible with downstream biochemical, quantitative LC-MS/MS and structural analyses (Figure 2A). This unique cross-linking approach depends on OGT’s catalytic activity and is expected to be more specific than many other reported methods, like photo-cross-linking. This is especially important considering that a few other enzymes, like EGF domain-specific O-GlcNAc transferase (EOGT)^63,64^ and protein GREB1 (ref 65.), have been recently reported to catalyze O-GlcNAcylation on proteins using OGT’s sugar donor, UDP-GlcNAc. Taken together, GEPs provide a set of powerful tools to accelerate the characterization of OGT-substrate binding and recognition. Another challenge in studying the role of OGT has been the paucity of inhibitors with sufficient potency and selectivity for cellular applications. Due to the high cellular concentration of UDP-GlcNAc (hundreds of micromolar range), many reversible active-site inhibitors of OGT are rendered less effective. Built on our successful targeting of OGT with GEPs, we developed a family of cell-permeable covalent inhibitors (Figure 2B).^66^ These inhibitors demonstrated significantly higher inhibition efficiency compared to reversible OGT inhibitors. More interestingly, we found that OGT was inhibited in cells with minimal effect on the functionally similar glycosyltransferase, EOGT, making this class of inhibitors an invaluable tool for targeting OGT active-site function in cells.

**Figure 2:**
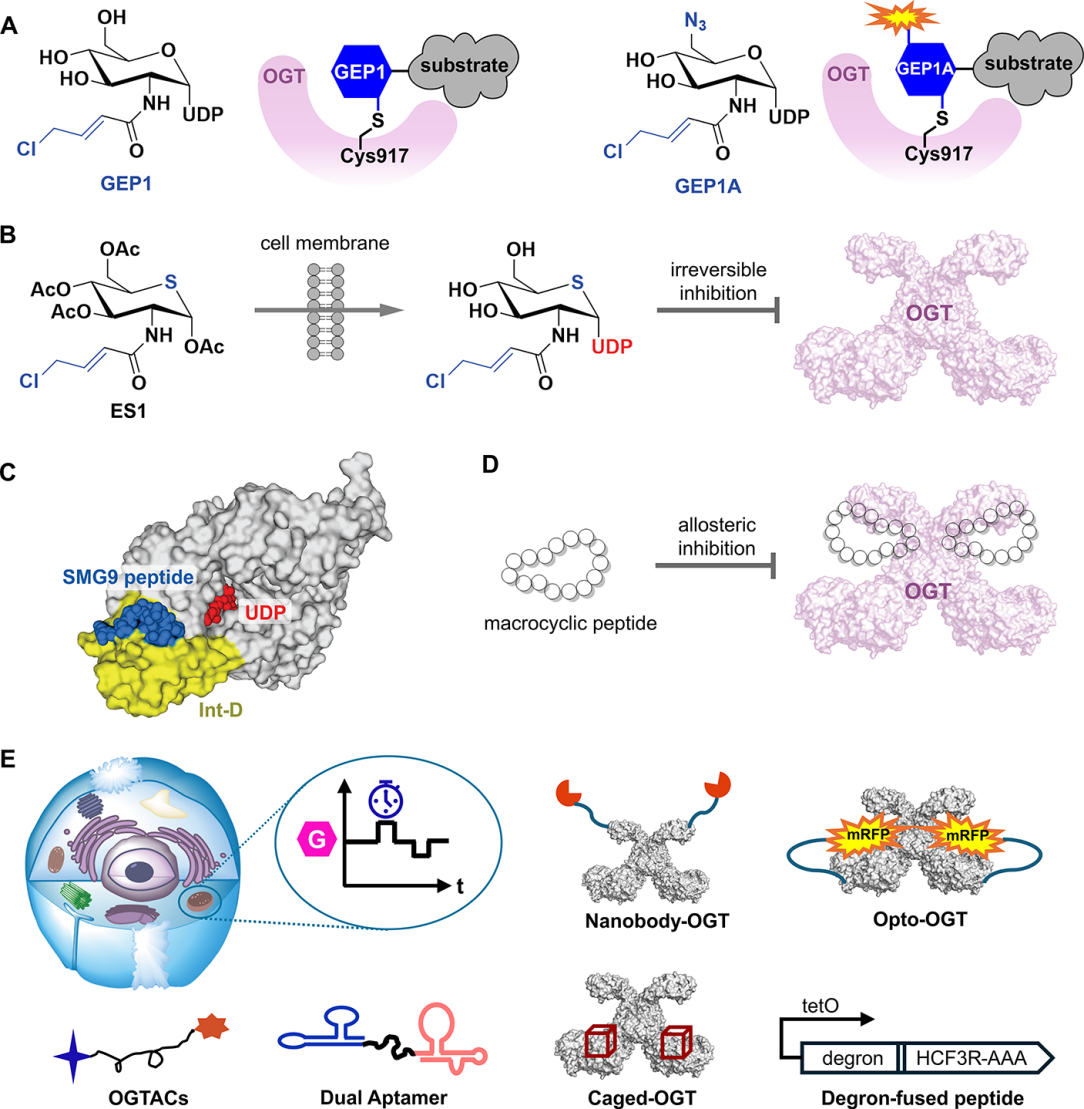
**A**. Representative GlcNAc Electrophilic Probes (GEPs) that can be applied to cross-link OGT with its substrates during the sugar transferring process and to investigate OGT–substrate binding modes. **B**. Targeted covalent inhibitor of OGT. **C**. Crystal structure of OGT-UDP-SMG9 peptide illustrates a conserved peptide motif binding to OGT Int-D (yellow) (PDB 8FE7). **D**. Inhibitory macrocyclic peptides targeting the TPR domain of OGT. **E**. Representative chemical biology tools targeting OGT for spatiotemporal control of O-GlcNAcylation inside of cells. Symbol G in pink hexagon: O-GlcNAcylation; t: time.

Previous studies from our group and others suggest that regions outside of OGT’s active site play critical roles in substrate binding, protein–protein interactions (PPIs), and cellular translocalization, to name a few.^15,33,40,41,62^ However, the specific sites of interaction modulating their functions remain largely undefined. Moreover, it is unclear if the nutrient sensing role of OGT relies solely on the catalytic site or an interplay between catalytic and non-catalytic regions. Further, it has been reported that the non-catalytic roles of OGT are necessary for mammalian cell proliferation, but the mechanisms remain unknown.^67^ Growing evidence suggests significant, previously unidentified roles for regions outside of the OGT’s active site. This encouraged us to explore the potential existence of remote binding sites for OGT interacting with substrate and nonsubstrate proteins. OGT is a unique glycosyltransferase that contains TPRs, a PPI scaffolding domain. Data from our group and others support that some proteins require TPR interactions for optimal O-GlcNAcylation, while other OGT substrates make minimal or partial TPR contact, suggesting additional, novel OGT binding sites that have not yet been investigated.^40,41,62^ Indeed, another notable feature of OGT is the Int-D (Figure 1B), whose function remains elusive, that is highly conserved in vertebrates, but its unique structural fold is not found in any other proteins. In a collaborative study, we have applied a Proteomic Peptide Phage Display (ProP-PD) screening technique and identified a novel peptide motif PxYx­[I/L] interacting with OGT.^42^ This motif exists in diverse human proteins, representing the first OGT specific short linear motif (SLiM) within the intrinsically disordered regions (IDRs) of binding proteins. Meanwhile, phage display with a random peptide library discovered a similar consensus sequence [Y/F]­xPxYx­[I/M/F] binding to OGT.^43^ Our crystal structures further reveal that the motif containing peptides bind to the OGT Int-D, distal from the active site (Figure 2C), providing the first direct evidence for the biological roles of this cryptic Int-D domain and revealing a novel OGT regulatory site. In the crystal structure of OGT_4.5_ in complex with an SMG9 peptide containing the motif, we found that the Int-D pocket contains several well-conserved residues in vertebrates, such as I734, I787, and N791 which make critical interactions with the SMG9 peptide in the structure (PDB 8FE7). Mutagenesis further validated the novel binding mode. Moreover, mutation of SMG9 at the motif Y residue, corresponding to Y147F mutation in the SMG9 protein, significantly reduced the interaction of the SMG9 peptide with OGT. SMG9 protein is a critical player in nonsense-mediated mRNA decay (NMD), targeting premature stop codon-containing mRNAs for rapid degradation.^68^ It is essential for mammalian embryonic and brain development, as well as modulation of the stress response.^69^ It has been reported that the SMG9 protein contains a single major O-GlcNAcylation site on residue T114, only 30 aa upstream of the Int-D binding motif. Remarkably, structure-informed mutation of OGT-N791A or SMG9-Y147F significantly impeded their association and O-GlcNAcylation of the SMG9 protein. A working model is that OGT TPRs may serve as a general scaffold for various protein partners, while the Int-D may serve as an anchor point for specific interactions with proteins via a binding motif, orienting appropriate Ser/Thr residue in the active site for O-GlcNAcylation. Interestingly, the catalytic activities of wild-type (WT) OGT and the N791A mutant are comparable, as evaluated using the well characterized CKII peptide substrate in vitro.^42^ These results, along with cellular studies, consistently support that the Int-D binding site does not affect OGT’s intrinsic catalytic activity, but instead regulates OGT-protein interactions and O-GlcNAcylation of OGT substrates like SMG9. More intriguingly, the SMG9 Y147 residue is a major phosphorylation site, which our structure-guided molecular dynamics (MD) simulation predicted would abolish interaction with OGT. Indeed, phosphorylation of Y147 in the motif of SMG9 disrupted its interaction with OGT in vitro.^42^ Since OGT has been proposed as a critical “nutrient sensor”,^22,48^ we tested global O-GlcNAcylation at different nutrient levels from cells that inducibly express WT OGT or the N791A mutant (abolishes motif binding to Int-D). In high nutrient conditions, both WT OGT and N791A mutant cells demonstrated similar levels of cellular O-GlcNAcylation. Strikingly, in low nutrient conditions, the N791A mutant cells failed to promptly downregulate O-GlcNAcylation after 48 h of nutrient starvation, despite similar OGT and OGA protein levels in both cells.^42^ Notably, we detected significantly lower levels of lactate production in N791A mutant cells with similar growth rates as WT OGT cells, supporting that the OGT Int-D site regulates glucose metabolism in cells. Collectively, these results suggest that the OGT Int-D site regulates cellular response to nutrient stress, but the molecular mechanisms require further investigation. Exciting discoveries of the OGT TPR and Int-D as functional regulators will promote the development of novel chemical tools to target these non-catalytic regions for site-specific modulation. As an example, highly potent macrocyclic peptides targeting the OGT TPR domain have been recently identified through a random nonstandard peptides integrated discovery (RaPID) system (Figure 2D).^70^ Further optimization of these macrocyclic peptides and new probe development for the Int-D can potentially lead to novel allosteric modulators to investigate OGT functions.

Spatial and temporal control of OGT function is essential for understanding its role in various cellular processes. Recent developments have provided valuable tools to help achieve this goal (Figure 2E). For instance, aptamers targeting OGT’s TPR region have been identified from a Systematic Evolution of Ligands by EXponential Enrichment (SELEX) screening platform.^71^ A dual aptamer with two functional domains, one binding to OGT and the other binding to a substrate protein, enables targeted O-GlcNAcylation of that specific substrate. Another recent strategy for targeted O-GlcNAcylation is the development of heterobifunctional small molecules called O-GlcNAcylation TArgeting Chimeras (OGTACs).^72^ In this strategy, FKBP12^F36V^ is fused to OGT as a binding handle, and a small molecule ligand of FKBP12^F36V^ is linked with another small molecule ligand of the targeted OGT protein substrate(s). The OGTAC brings together OGT and its substrates to induce protein-specific O-GlcNAcylation in cells. Aside from aptamers and small molecules, engineered OGT through fusion to a nanobody was also applied to targeted O-GlcNAcylation.^73^ The nanobody recognizes the substrate and brings OGT in proximity to induce O-GlcNAcylation. In a different strategy, a photoactivatable OGT was constructed through a genetic codon expansion technique by replacing an essential catalytic lysine (K842) with a genetically encoded photocaged lysine analog.^74^ Upon near UV (365 nm) irradiation, the protecting group on K842 was released and exposed the caged OGT, enabling its catalytic function. This strategy showed that activation of OGT could reverse the morphological contraction of fibroblasts, linking dynamic O-GlcNAcylation to cellular contractile responses. Through engineering a short form of OGT, which is located in the cytosol, this photoactivatable method demonstrated its spatial activation at a subcellular level. More recently, an Opto-OGT is created by fusing a full-length human OGT with mCherry and the photosensitive cryptochrome 2 (CRY2) domain.^75^ In the dark, CRY2 is proposed to sterically hinder the UDP-GlcNAc substrate binding site of OGT. Upon exposure to blue light, CRY2 undergoes conformational changes and oligomerizes, thereby exposing OGT and permitting substrate access. Opto-OGT is demonstrated to be reversible, tunable, and robust through engineering. By coupling Opto-OGT with targeting motifs via the heterodimerization system between CRY2 and its interaction partner CIBN, OGT is redirected to specific subcellular locations such as the mitochondria and plasma membrane. Recently, the exceptional binding affinity of HCF-1 peptide (a natural ligand of OGT) has been leveraged to develop a degradable and switchable OGT inhibitor for cellular use.^76^ Upon doxycycline induced expression of a fluorescently tagged HCF-1 peptide variant (HCF3R-AAA peptide fused with FKBP12^F36V^), OGT’s activity can be inhibited by this engineered peptide. Interestingly, the inhibition effect can be reversed by the treatment of dTAGV-1, which is a degrader targeting the FKBP12^F36V^ fused HCF3R-AAA peptide. This switchable system can be used to understand the dynamic regulation of O-GlcNAc levels. Taken together, these novel chemical biology tools represent significant advancements in interrogating and manipulating the specific interactions and functions of OGT in vitro and in cells.^71–76^

## 3 O-GlcNAcase (OGA)

### 3.1 A Brief Overview of O-GlcNAcase (OGA)

O-GlcNAcase (OGA), the sole enzyme that removes O-GlcNAc modifications, consists of multiple domains: an N-terminal catalytic domain, a stalk domain, and a C-terminal pseudo histone acetyltransferase (pHAT) domain^77^ (Figure 3A). Two main isoforms of OGA have been identified: full-length OGA (916 aa) and a short OGA (677 aa) lacking the pHAT domain.^78^ This Account will focus on the more abundant isoform, full-length OGA, and highlight the advances in its structural studies to promote chemical biology developments. Given the variety of important biological functions and diversity of OGA substrates, elucidating OGA’s structure, understanding how different protein substrates bind OGA, identifying what the critical substrate recognition elements are, and determining if interactions can be modulated specifically is paramount. The existence of multiple intrinsically disordered regions (IDRs, including the N-terminal tail (1–59 aa), the stalk domain loop (400–552 aa), and the loop bridging the stalk domain and pHAT domain (705–715 aa)) poses a significant challenge for characterization of the full-length OGA structure. We and others have determined the X-ray crystal structures of truncated OGA constructs (e.g., OGA_cryst_ in Figure 3A,B), which comprise the catalytic and stalk domains without the IDRs.^17–19^ These structures offer the first view of human OGA, providing a model to elucidate OGA structural features for catalysis and substrate recognition. OGA’s catalytic site folds into a classic (β/α)_8_-barrel (eight-stranded parallel β-sheet core mainly surrounded by eight α-helices), which forms a deep pocket for GlcNAc binding and hydrolysis. The OGA stalk domain folds into an α-helix bundle. Distinct from many bacterial OGA homologues in the GH84 family, human OGA forms an arm-in-arm dimer with the stalk domain of one monomer covering the catalytic domain of the other, forming a cleft for potential substrate/protein binding. Our structural analyses of OGA_cryst_ in complex with different O-GlcNAcylated peptides further revealed unique substrate binding modes^19,79^ (Figure 3B). These structures illustrate that GlcNAc binding in the catalytic pocket is highly conserved, while the peptide part of the O-GlcNAcylated substrates can potentially make different interactions with OGA stalk domain residues, contributing to OGA’s substrate recognition and discrimination. These OGA structural complexes with distinct glycopeptides, along with the growing evidence from biochemical and cellular studies, suggest that in addition to the catalytic site, the non-catalytic domains (e.g., stalk domain) can also contribute to OGA substrate recognition. This new perspective is further supported by our recent study on a cancer-derived mutant of OGA.^80^ We found that a single mutation (S652F) on the non-catalytic stalk domain of OGA dysregulated its interaction with a set of specific substrate and nonsubstrate proteins. One of the most dysregulated substrates is PDZ and LIM domain protein 7 (PDLIM7). Interestingly, compared to the WT enzyme, the OGA mutant more favorably removed the O-GlcNAc modification from PDLIM7 protein, significantly reduced the level of p53 tumor suppressor and elevated actin-rich membrane protrusions to promote cancer cell motility and aggressiveness.^80^ These findings illuminate the critical role of a stalk domain mutation in dysregulating OGA’s substrate specificity to promote cell malignant progression, opening new opportunities for chemical biology development in anticancer research. More recently, two studies have reported the cryo-EM structures of a human OGA dimer containing pHAT domains: one is full-length OGA,^20^ while the other is a multidomain OGA lacking the IDR spanning residues 397–535 (ref 21.). Although a displaced helix was observed on one of the full-length OGA chains in the cryo-EM structure,^20^ in both studies, the positioning of catalytic residues and the overall fold of the catalytic and stalk domains of each OGA monomer closely resembles that of the published truncated OGA crystal structures (e.g., PDB 5VVO). While a high-resolution structure of the human OGA pHAT domain was not attained, these cryo-EM data consistently support the substantial flexibility of pHAT domains relative to the catalytic-stalk dimer, providing novel insights into OGA’s substrate recognition and functional regulation (see below).

**Figure 3:**
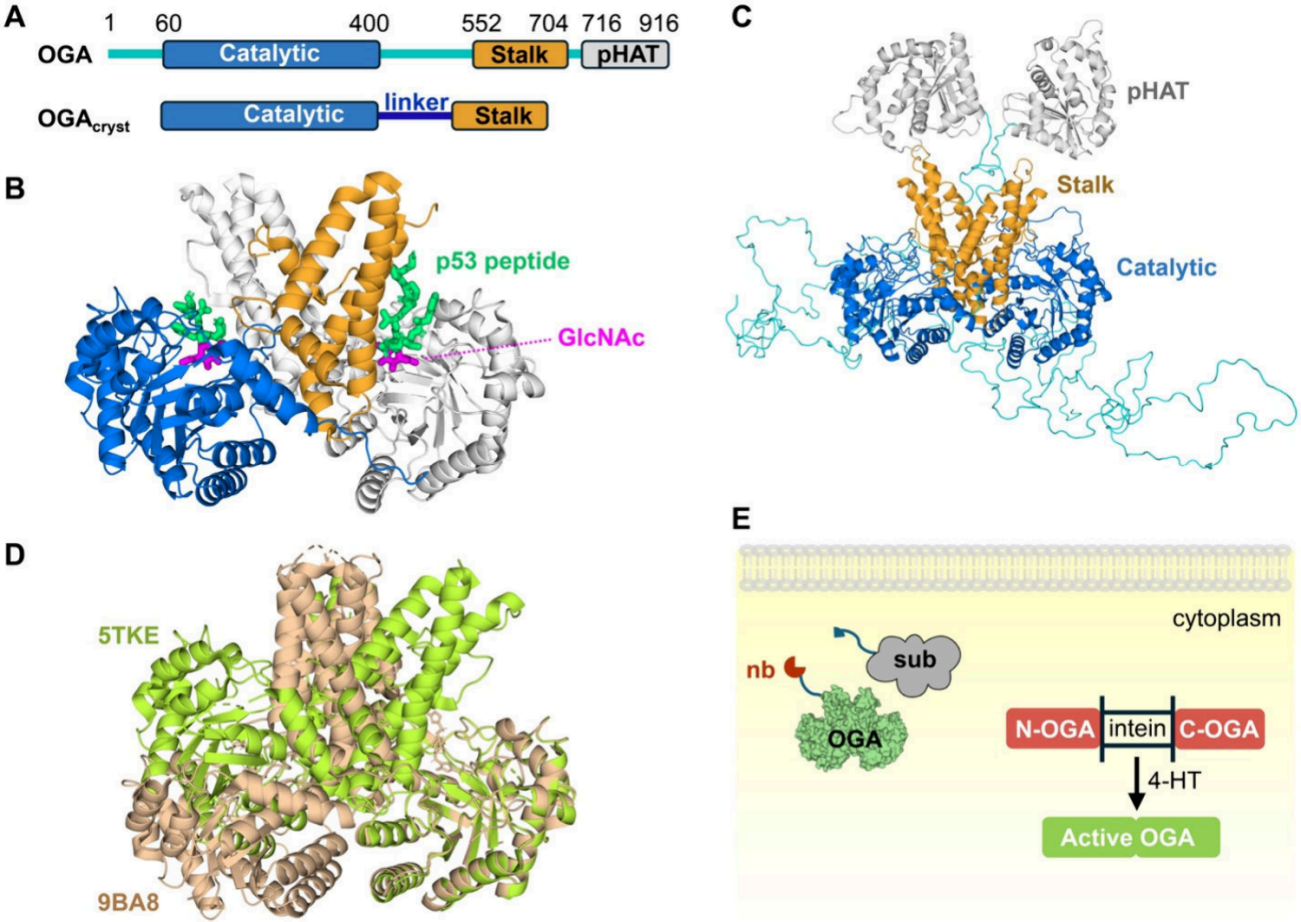
**A**. Domain architecture of human O-GlcNAcase (OGA) and a crystallization construct (OGA_cryst_). **B**. The crystal structure of OGA_cryst_ dimer in complex with O-GlcNAcylated p53 peptide substrate (PDB 5UN8). The catalytic and stalk domains of one OGA_cryst_ monomer are colored in blue and orange, respectively, while the sister OGA_cryst_ monomer is shown in gray. The O-GlcNAcylated p53 peptide is colored in pink (GlcNAc) and green (peptide). **C**. Full-length OGA model rebuilt from an AlphaFold3 prediction (ref 29). **D**. Superimposition of two different crystal structures of OGA catalytic-stalk domain dimers (Forest: apo form (PDB 5TKE); Wheat: small molecule inhibitor bound form (PDB 9BA8)). **E**. Representative chemical biology tools targeting OGA for spatiotemporal O-GlcNAc hydrolysis. Sub: substrate; Nb: nanobody; 4-HT: 4-hydroxytamoxifen.

### 3.2 The Structural Flexibility of OGA

While full-length OGA has been investigated structurally,^20,21^ the high-resolution model is currently only accessible by prediction (Figure 3C). Previously reported crystal structures of truncated OGA (e.g., OGA_cryst_ in Figure 3B) and their glycopeptide complexes typically display a partially “open scissor” shaped dimer conformation.^17–19^ It was hypothesized that the OGA protein may possess some flexibility to alter (e.g., open or close) the dimer conformation for accommodating various substrates and binding partners. Intriguingly, a crystal structure (PDB 9BA8, Figure 3D) of truncated OGA complexed with an active-site inhibitor displayed a “closed scissor” conformation with each monomer retaining the classic fold as mentioned above.^81^ While it remains to be determined whether this atypical dimer conformation arises from crystal packing, is induced by inhibitor binding, or reflects a ligand-stabilized state via conformational selection within a dynamic landscape, the observation suggests that the OGA dimer core is not rigid. This hypothesis is in line with the full-length OGA cryo-EM structural model. It is likely that the OGA dimer core could be open or closed at varying levels to accommodate distinct binding partners. Nevertheless, the ligand properties that regulate such conformational changes are unclear. Also, it would be interesting to evaluate if OGA conformational changes can be exploited for future development of novel allosteric modulators. Ideally, the design of a selective compound capable of modulating either the open or closed conformation of OGA would enable targeted regulation of enzyme’s activity toward specific protein substrates, thereby providing insights into associated biological functions. Compared to OGT, the significantly higher flexibility of OGA is attributed to the existence of multiple intrinsically disordered regions, such as the N-terminal tail (1–59 aa), the stalk domain loop (400–552 aa), and the loop bridging the stalk domain and pHAT domain (705–715 aa) (Figure 3A). The inherent flexibility of these regions has hindered high-resolution structural characterization of full-length OGA by both X-ray crystallography and cryo-EM. Recently, two studies reported low-resolution cryo-EM structural models of OGA (i.e., full-length OGA and a multidomain OGA without the stalk domain loop),^20,21^ demonstrating considerable dynamics of the pHAT domain (Figure 3C). This dynamic positioning of the pHAT domain, largely derived from the flexible loop bridging the stalk domain and pHAT domain, may have functional implications for OGA regulation. Indeed, the position of the pHAT domain can regulate the exposure of amino acids in the environment near the OGA active site, acting as a potential allosteric regulator of OGA.^21^ This arrangement likely provides a fine-tuned mechanism for modulating activity, possibly by influencing substrate recognition or enzyme dynamics. While the pHAT domain of OGA cannot bind acetyl-CoA and therefore does not possess acetyltransferase activity,^82^ it is likely that it retains the ability to interact with other proteins. Notably, emerging evidence suggests that the pHAT domain of OGA acts as a “reader” of histone modifications.^20^ Selective binding with both full-length OGA and its pHAT domain was detected for methylated and acetylated H3K36 and H4 histone tails. The pHAT domain may provide a flexible tether to recruit OGA enzymes to chromatin regions enriched in these modifications on histone H3 and H4, which are associated with transcriptional activation, transposon regulation, and DNA damage repair. These findings suggest that the dynamic nature and conformational plasticity of OGA are not merely structural features; they can play critical roles in modulating OGA’s substrate recognition, catalytic activity, and regulatory functions. Flexible structural elements may influence, for example, the access, recognition and orientation of substrates or binding partners. Conformational flexibility is also relevant for acquiring enhanced substrate promiscuity. Flexible regions in OGA may act as molecular switches or regulatory scaffolds, enabling interactions with diverse substrates or binding partners. Moreover, it would be interesting to explore if OGA’s flexibility profile is intimately linked to ligand properties or related mechanisms in ligand recognition/interaction. This information will lay an important foundation for future design of chemical or engineered tools to modulate OGA functions.

### 3.3 Post-translational Modifications (PTMs) of OGA

Both OGT and OGA can be modified by various post-translational modifications (PTMs).^46^ However, unlike many PTMs on OGT with specific functions investigated in vitro or in vivo, most PTMs on OGA (e.g., phosphorylation, O-GlcNAcylation, acetylation, formylation, SUMOylation, and ubiquitination) were only identified using proteomics approaches^45,46^ and their functional roles remain largely unexplored. Perhaps one of the most notable PTMs on OGA is its cleavage by caspase-3 at its D413 residue on the stalk domain loop.^83^ Intriguingly, the cleaved OGA remains catalytically active. Moreover, the single O-GlcNAc modification of OGA is located at its S405 residue, which is only a few amino acids away from the caspase-3 cleavage site. Following these interesting observations, many questions remain to be explored, such as whether there is any crosstalk between the O-GlcNAcylation and cleavage of OGA, what are the functional impacts of OGA cleavage, and how can OGA prevent the hydrolysis of its own O-GlcNAcylation? Addressing these questions is expected to yield new insights into the multifaceted mechanisms underlying OGA’s functional regulation. Very recently, SUMOylation at the K358 residue, located on the conserved internal flexible loop region of OGA, has been reported to mediate its binding with chaperone HSC70, promoting the degradation of OGA through chaperone-mediated autophagy.^84^ This new evidence supports the quantity control of OGA protein inside cells, but whether this PTM affects OGA’s activity and/or its substrate selectivity remains unknown. In addition to studying the roles of existing PTMs, there is a need to detect potentially novel PTMs on endogenous OGA proteins in tissue samples and various conditions. As a chemical strategy to help address this need, a class of OGA inhibitors (called bicyclic thiazolidines) was developed to facilitate the enrichment and identification of new PTM types and sites (e.g., O-GlcNAcylation, acetylation, and formylation) on endogenous OGA.^85^ The crystal structures of OGA in complex with these inhibitors have revealed that the bound compounds extend outside of the enzyme’s catalytic pocket, allowing functionalization to develop high affinity chemoproteomic probes for the purification of endogenous OGA from brain tissue. These new probes enable tissue-specific detection of PTMs on endogenous OGA, emphasizing the importance of chemical tools for the study of OGA PTMs and their roles in vivo.

### 3.4 Chemical Biology Tools for OGA

Significant efforts in the past few years have led to the development of a number of small-molecule inhibitors and engineered OGA constructs as powerful tools to modulate OGA activity in vitro and in cells.^81,85–87^ These studies provided critical insights into the functional roles of OGA and dynamic O-GlcNAcylation. Currently, most OGA inhibitors are designed to competitively block the enzyme active site, which can effectively maintain elevated O-GlcNAc levels on a global scale. To achieve protein-specific O-GlcNAc hydrolysis, a split OGA construct was fused to a nanobody that can recognize a particular protein substrate^86^ (Figure 3E). This engineered OGA, along with engineered OGT, enables reversible and protein-selective editing of O-GlcNAc levels in living cells. In another design, a truncated form of OGA, lacking the pHAT domain and fused to an evolved intein, which is responsive to the small molecule 4-hydroxytamoxifen (4-HT), can induce O-GlcNAc hydrolysis in cells with potential spatial and temporal precision^87^ (Figure 3E). In the absence of 4-HT, the OGA–intein fusion is inactive. Upon 4-HT treatment, the intein undergoes self-splicing, releasing a spliced, active form of OGA to induce O-GlcNAc hydrolysis. Dose- and time-dependent activation of OGA can be achieved by varying the amount and treatment duration of 4-HT. By further fusing subcellular targeting signals, this engineered OGA can restrict its deglycosylation activity to specific cellular compartments, thereby allowing spatial control of O-GlcNAc removal. With the recent advancement in OGA structural and biochemical studies, targeting OGA’s noncatalytic domains (e.g., stalk domain and pHAT domain) or their interactions can potentially offer novel approaches to modulate OGA function toward specific endogenous proteins while preserving its basal catalytic activity to maintain global O-GlcNAc homeostasis.

## 4 Summary and Perspectives

OGT and OGA are a single pair of human enzymes controlling O-GlcNAc dynamics on thousands of proteins involved in numerous biological processes.^1^ Dysregulation of these enzymes can lead to various pathological conditions, including cancer, diabetes, and neurodegenerative disorders.^23–25,28^ One of the most intriguing questions is how these enzymes recognize their substrates and regulate their multifaceted roles in response to nutrients and stress. This Account highlighted recent progress in studying these essential enzymes, especially focusing on their structural studies with implications for functional investigation and chemical biology development. In the past decade, remarkable progress has been achieved on the structures of OGT and OGA proteins, as well as a series of innovative chemical and engineered tools that can inhibit or induce the activities of these enzymes.^15–19,31,56,58–60,88^ However, our understanding of their mechanisms of regulation is far from complete. This is in part because previous studies have been mainly focused on the well folded regions, especially the catalytic domains, of these enzymes, while noncatalytic regions have received comparatively little attention. Emerging evidence supports that “functional hotspots” can be found outside of the enzyme active site, including OGT’s TPR region and intervening domain (Int-D), and OGA’s stalk domain and pHAT domain. These O-GlcNAc cycling enzymes possess unique structural features that may underlie their mechanisms of substrate recognition and functional regulation. Moreover, structural flexibility and post-translational modifications (PTMs) further complicate the regulatory landscape of these enzymes. Beyond protein-level regulation, OGT and OGA are also subject to control at the RNA level. For instance, splicing silencers can modulate OGT expression,^89^ while detained intron retention has been implicated in the regulation of both OGT and OGA transcripts.^90^ Collectively, these findings highlight the multilayered regulatory mechanisms that enable cells to fine-tune O-GlcNAc homeostasis in response to dynamic physiological conditions.

Several promising directions emerging from O-GlcNAc studies warrant further investigation. One of them is the mechanisms by which OGT functions in nutrient sensing.^22^ Despite decades of observations that OGT acts as a “nutrient sensor” in biology and disease, little is known about the molecular mechanisms underlying this essential function. An early study suggests that a putative phosphatidylinositol 3,4,5-trisphosphate (PIP3) binding site on OGT facilitates its recruitment to the plasma membrane during insulin signaling.^91^ This finding implies that metabolites may regulate OGT’s activity and subcellular localization, thereby linking nutrient sensing to O-GlcNAcylation. Recent work from our group and others has identified the intervening domain (Int-D), a poorly understood protein fold found only in OGT and its vertebrate homologues, as a motif-dependent regulator of OGT-protein interactions and O-GlcNAcylation.^42,43^ This novel binding site, along with its potential role in nutrient sensing, represents an exciting avenue for future research.^42^ A better understanding of OGT-protein interactions, particularly through regions beyond the OGT catalytic site, would be highly impactful, and will provide critical insights into 1) O-GlcNAcylation site selectivity, 2) allosteric regulation mechanisms, 3) the multifunctional roles and precise functional control of OGT, and 4) its dysregulation in disease. OGT is a master regulator with a remarkably broad range of substrate and nonsubstrate binding partners. It has evolved distinct structural features that enable the coordination of diverse modules, supporting the high plasticity and complexity of regulatory networks. Elucidating OGT’s novel binding modes is expected to not only facilitate the construction of a functional model for this essential enzyme, but also provide valuable insights into the regulation of its numerous interactors. This is particularly relevant to the poorly understood intrinsically disordered regions (IDRs) that interact with OGT, which are enriched in post-translational modifications (PTMs) and frequently dysregulated in disease. The findings discussed here will pave the way for selectively targeting OGT-mediated protein–protein interactions (PPIs) at novel regulatory sites. OGT TPR- and Int-D-mediated recognition of IDRs helps explain why most O-GlcNAcylation sites are found within these regions. However, OGT is also known to modify proteins with well-structured domains, where target residues are not readily accessible. How OGT recognizes and modifies these structurally constrained sites remains an open question. One possibility is that O-GlcNAcylation occurs cotranslationally on prefolded nascent peptides.^92,93^ Nevertheless, the molecular principles by which OGT accommodates such sites remain elusive and warrant further investigation.

Another interesting area for future research is the potential role of OGA’s pHAT domain. Emerging evidence suggests that this domain may bind to substrate or nonsubstrate proteins, facilitating the positioning of O-GlcNAcylated regions in proximity to the OGA active site for efficient deglycosylation.^94^ The use of remote binding sites may strengthen OGA-substrate interactions, improve catalytic efficiency, and enhance substrate selectivity.^20,21^ This mechanism may represent a means of long-range coordination in OGA’s substrate recognition and functional regulation. Furthermore, the hypothesis that the pHAT domain acts as a “reader” of histone modifications is interesting.^20^ This aligns with previous studies indicating that the pHAT domain serves as an interaction hub, regulating protein O-GlcNAcylation during DNA damage repair and potentially contributing to histone acetylation.^94,95^ Future investigations in this area are expected to provide significant insights into the epigenetic functions of OGA and its broader regulatory roles.

The intrinsic flexibility of O-GlcNAc cycling enzymes is not merely a structural feature, but a fundamental aspect of their substrate recognition and regulatory roles. Understanding the dynamic nature of these enzymes has broad implications for fundamental research on O-GlcNAcylation and the development of targeted therapeutics. However, probing protein flexibility using a single structural biology approach (e.g., X-ray crystallography or cryo-EM) is very challenging, particularly when the regions exhibit high conformational dynamics or lack defined secondary structure. It is expected that integrating interdisciplinary experimental techniques with computational modeling will offer new opportunities to address these challenges. For instance, nuclear magnetic resonance (NMR) spectroscopy is uniquely suited to capture dynamic motions at atomic resolution. While conventional NMR is generally limited by the target size, new developments have enabled the use of this technique for large proteins or complexes, such as nucleosome-binding proteins and ribonucleoproteins.^96–98^ It is expected that NMR can potentially be applied to investigate the conformational dynamics of OGT (or OGA) substrate complexes, especially considering that a methyl labeling approach can selectively label methyl groups on residues like Ala, Val, Leu, Ile, Thr, and Met, which are abundant in OGT and OGA.^96^ In addition, a segmental isotopic labeling approach, when coupled with expressed protein ligation and other chemical biology strategies, can label specific protein domains while leaving the rest of the protein unlabeled.^99,100^ This approach has been used effectively in kinase studies and can potentially be adapted to investigate the flexible regions in OGA, providing new insights into the dynamic behavior of this enzyme. In addition to NMR, small-angle X-ray scattering (SAXS) is another widely used approach to gain ensemble structural information on proteins in solution.^101^ SAXS data retain the native conformational heterogeneity of flexible proteins, and computational techniques such as the Ensemble Optimization Method (EOM) allow for detailed analysis of conformational ensembles.^102,103^ SAXS is especially powerful for dissecting the dynamic properties of OGA’s intrinsically disordered regions and OGT’s TPR interactions. Moreover, Single-Molecule Förster Resonance Energy Transfer (sm-FRET) enables real-time tracking of conformational changes in protein complexes, offering a complementary approach to study protein dynamics.^104^ By measuring energy transfer efficiency between fluorophore-labeled residues, sm-FRET can potentially capture dynamic transitions in OGT (or OGA) during ligand binding. This approach is particularly suitable for studying the impacts of protein flexibility in substrate recognition and other binding events. Integrating the data from complementary experimental approaches with computational modeling (e.g., molecular dynamics (MD) simulation) is expected to provide unprecedented details of the conformational dynamics for OGT and OGA. Such integrative approaches are essential for uncovering the molecular mechanisms of protein flexibility in facilitating substrate recognition and functional regulation.

As the field progresses, it has become evident that other regions of OGT/OGA beyond the catalytic domain play important functional roles.^33^ It is likely that different classes of proteins bind OGT or OGA using different binding modes, comprising a set of distinct regulatory sites instead of a single binding site in the enzyme’s catalytic pocket. Such diverse binding modes may underlie the divergent physiological and pathological functions of O-GlcNAc cycling enzymes, emphasizing the need for additional structures of these enzymes in complex with their substrates or nonsubstrate binding partners. Hence, it is important to continue studying these complex enzymes to better understand how they recognize their targets in terms of their promiscuity, specificity, and distinct preferences. In the meantime, other factors such as post-translational modifications and cellular localization can also contribute to the dynamic regulation of these O-GlcNAc cycling enzymes. Integrating information from different studies will facilitate the development of OGT/OGA modulators to enable more specific functional control and potential treatment of disease. It is expected that the studies of these unique enzymes will continue to surprise us in the future.
